# Supplementary figures and images for: CryptoCEN: A Co-Expression Network for Cryptococcus neoformans reveals novel proteins involved in DNA damage repair
Source: PLoS Genet. 2024 Feb 15;20(2):e1011158. doi: 10.1371/journal.pgen.1011158 (PMC10901339; doi:10.1371/journal.pgen.1011158)

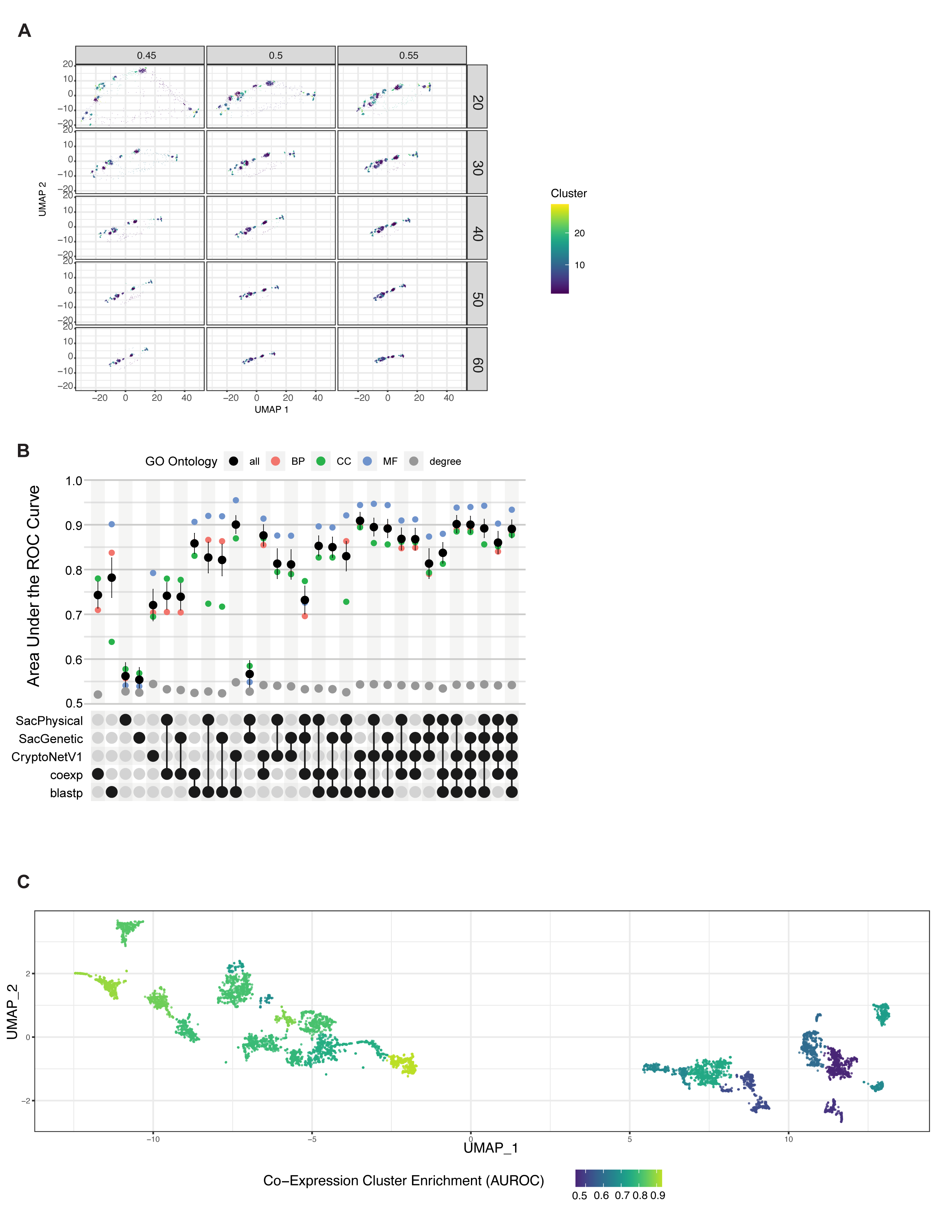

Supplement: S1 Fig — A. A re-embedded gene by expression matrix, scanning the key embedding parameters umap_a and umap_b over the ranges [20, 30, 40, 50, 60] and [0.45, 0.5, 0.55], respectively, while keeping the remaining parameters fixed (prereduction of dimension to 500 using PCA, n_neighbors = 30, negative_sample_rate = 50, umap_repulsion_strength = 3, n_epochs = 2000). Rows are umap_a and the columns are umap_b. The points are clustered using leiden clustering using with a resolution parameter of 1e-3 and points are colored by the cluster index. B. UpSet plot of the retrospective prediction accuracy, as determined by the neighbor voting guilt-by-association (GBA) area under the ROC curve (AUROC). AUROCs values range between 0.5 for random predictor and 1 for a perfect predictor. As data sources are combined, the prediction accuracy increases. Each annotated GO term is colored by ontology biological process (BP), cellular component (CC), or molecular function (MF). C. Enrichment of co-expression in the gene by expression matrix UMAP. For each cluster, we selected inter and intra-cluster associations. We then computed the area under the receiver operator characteristic (AUROC) for the enrichment of the intra-cluster associations over the inter-cluster associations based on the co-expression score. Enrichment within each cluster is indicated by color. (TIF) [file pgen.1011158.s001.tif]

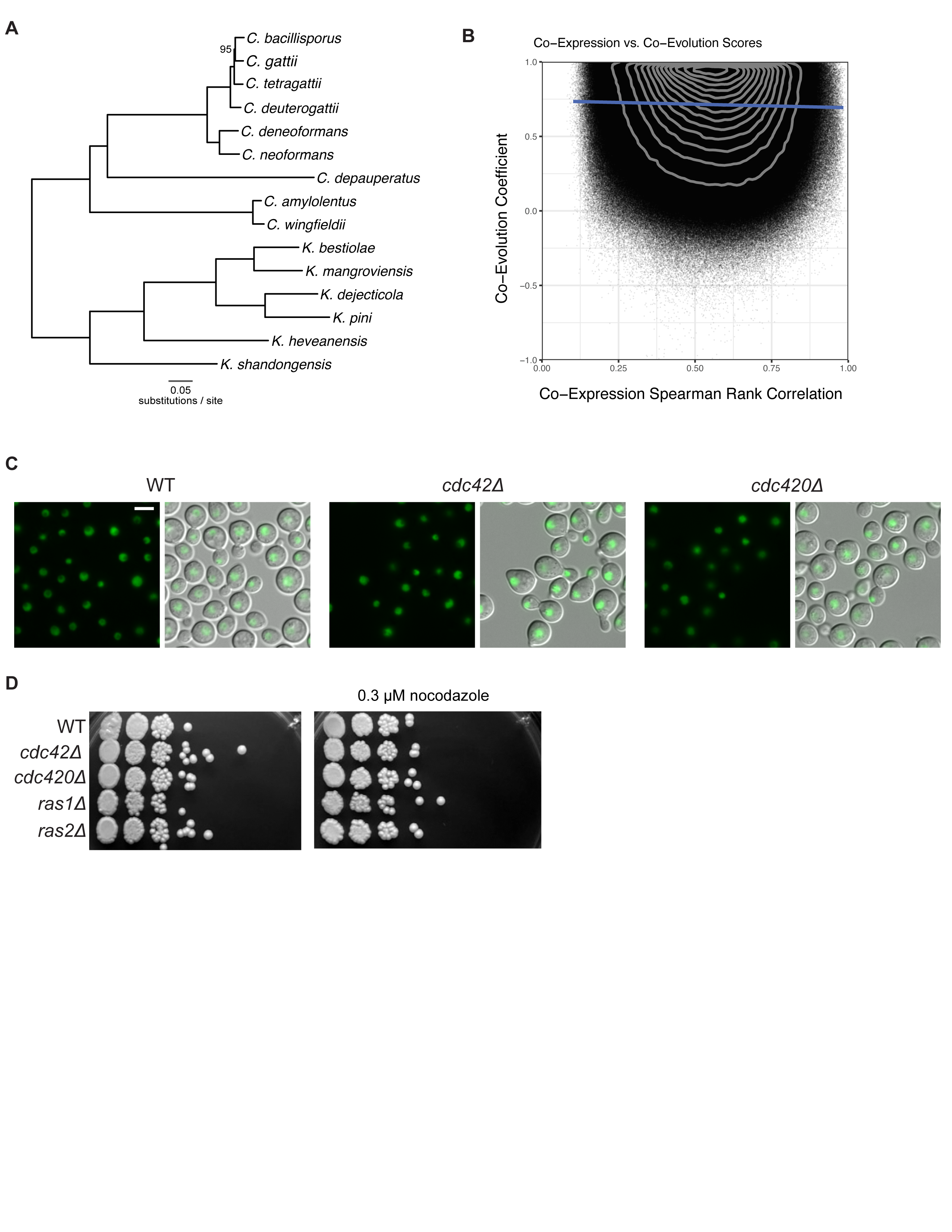

Supplement: S2 Fig — A) Phylogeny used for co-evolution. Tips indicate each species, scale bar indicates 0.05 substitutions/site. B) Co-expression vs. co-evolution scores over the 5,264 overlapping gene sets does not show a positive correlation (correlation coefficient is -0.001). C) There is no difference in histone localization between the strains. Histones were marked by fusion of H4 with GFP. Cells were incubated in liquid YPD at 30°C before imaging. Images taken at 40X magnification, scale = 5 microns. D) There is no difference in nocodazole sensitivity between the cdc42Δ or cdc420Δ mutant strains. The indicated strains were grown overnight at 30°C in liquid YPD medium and then serially diluted onto YPD or plates containing 0.3 μM nocodazole and incubated for 2 days before imaging. (TIF) [file pgen.1011158.s002.tif]

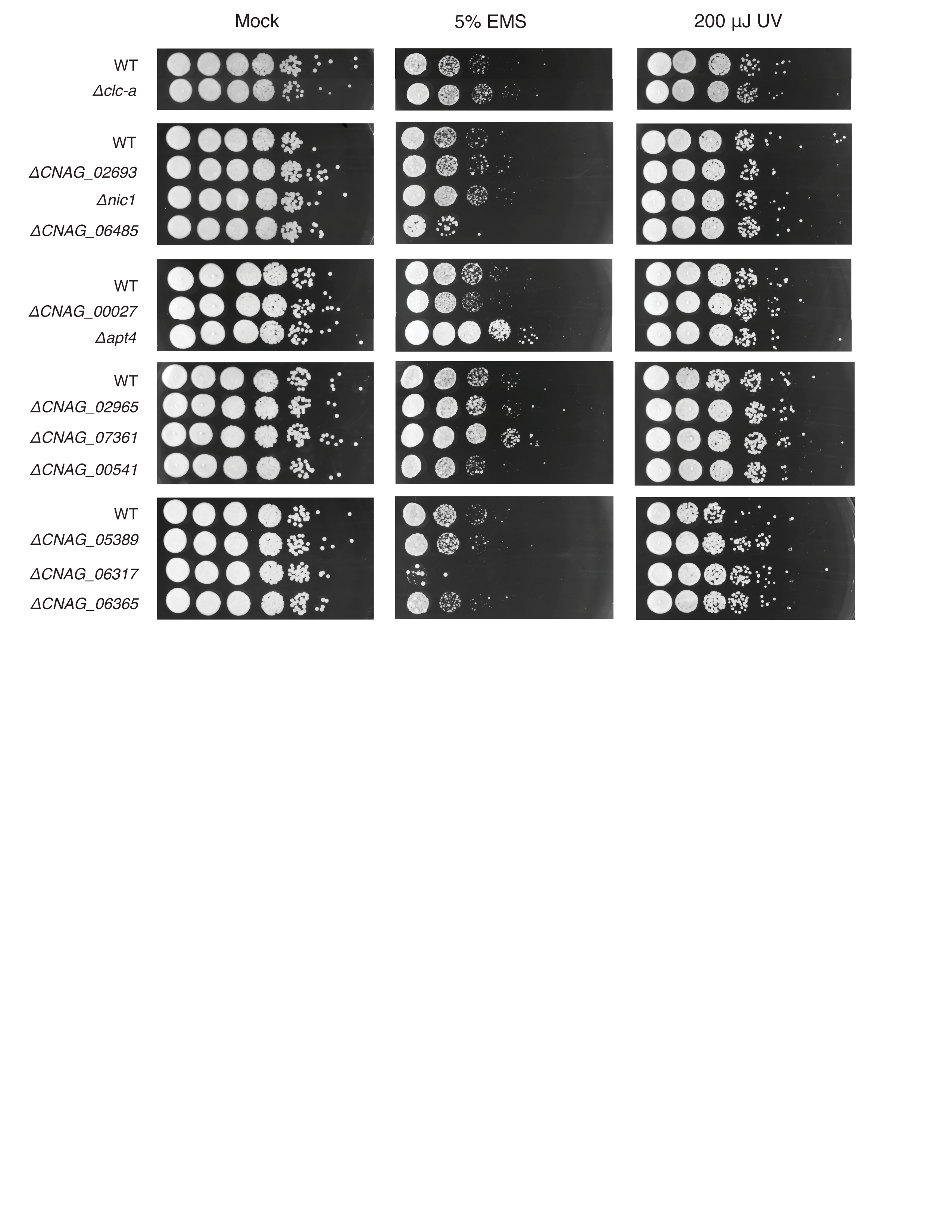

Supplement: S3 Fig — A) Analysis of matched controls for phenotypes on DNA damaging agents. The indicated strains were grown overnight at 30°C in liquid YPD medium, and the 10-fold serially diluted cells were spotted onto YPD agar. For UV damage, the plates were immediately subjected to 200 μJ UV. For EMS, the cells were incubated in 5% EMS for 1 hr before serial dilution and plating. The plates were incubated at 30°C and imaged after 2 days. (TIF) [file pgen.1011158.s003.tif]

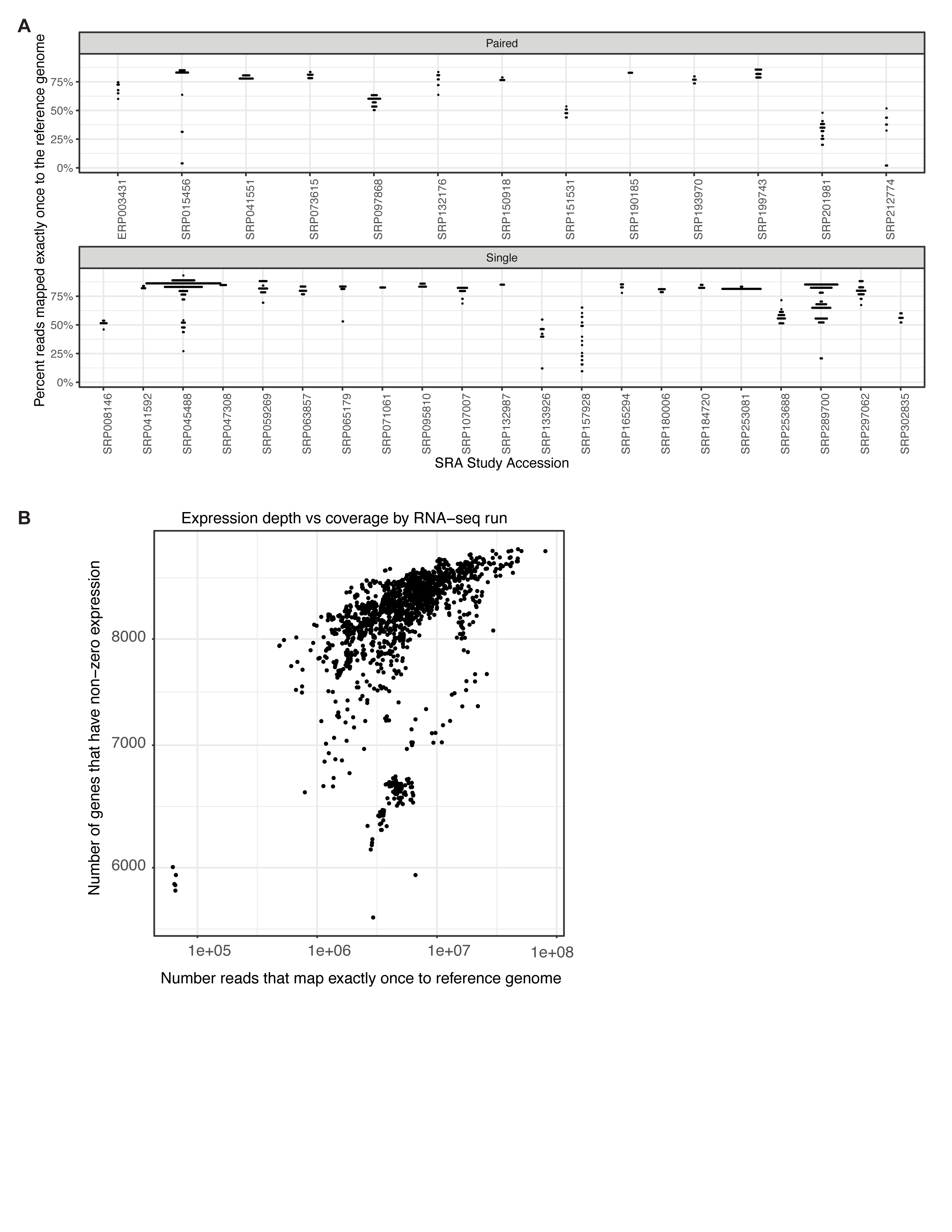

Supplement: S4 Fig — A. A total of 1,523 RNAseq runs from 34 identified studies are scatter-plotted as the number of genes with nonzero expression versus the fraction transcripts that map exactly once. B. The RNAseq runs that have nonzero expression for at least half of the genes which are used to construct the CryptoCEN network. (TIF) [file pgen.1011158.s004.tif]

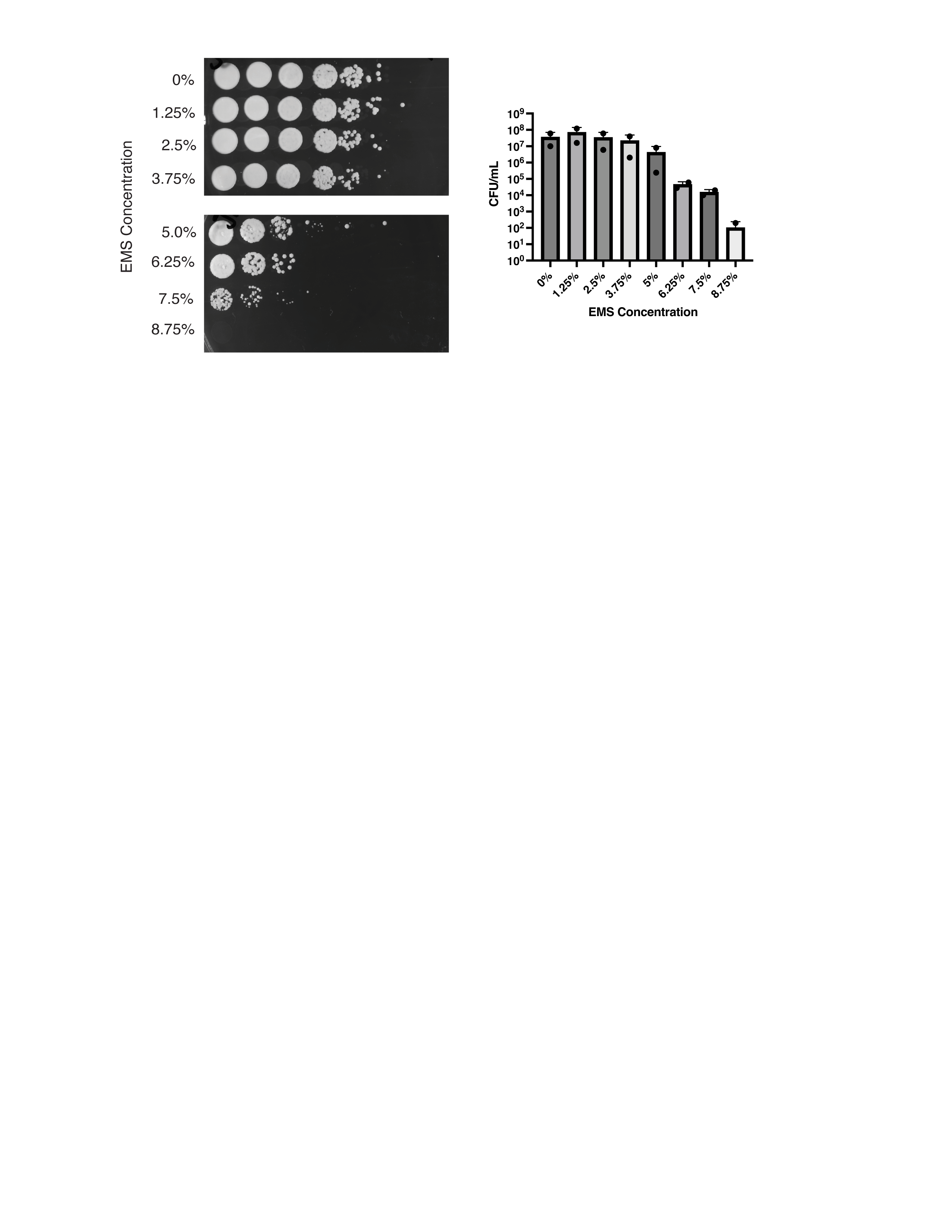

Supplement: S5 Fig — A) H99 wild type cells were incubated with the indicated concentrations of EMS for 1 hr before serial dilution and plating onto YPD. The plates were incubated at 30°C and imaged after 2 days. CFU/mL was calculated from the serial dilutions. (TIF) [file pgen.1011158.s005.tif]
